# Unraveling the Complexity of Parkinson's Disease: Insights into Pathogenesis and Precision Interventions

**DOI:** 10.1002/advs.202405309

**Published:** 2024-09-20

**Authors:** Han Yan, Cole Coughlin, Lee Smolin, Jin Wang

**Affiliations:** ^1^ Wenzhou Institute University of Chinese Academy of Sciences Wenzhou 325001 P. R. China; ^2^ Perimeter Institute for Theoretical Physics 31 Caroline Street North, Waterloo Ontario N2J 2Y5 Canada; ^3^ Department of Chemistry and Physics State University of New York at Stony Brook Stony Brook NY 11790 USA

**Keywords:** compensatory mechanisms, intervention, non‐equilibrium landscape and flux, parkinson's Disease

## Abstract

Parkinson's disease (PD) is a neurodegenerative disorder characterized by dopaminergic neuron loss, leading to motor and non‐motor symptoms. Early detection before symptom onset is crucial but challenging. This study presents a framework integrating circuit modeling, non‐equilibrium dynamics, and optimization to understand PD pathogenesis and enable precision interventions. Neuronal firing patterns, particularly oscillatory activity, play a critical role in PD pathology. The basal ganglia network, specifically the subthalamic nucleus‐external globus pallidus (STN‐GPe) circuitry, exhibits abnormal activity associated with motor dysfunction. The framework leverages the non‐equilibrium landscape and flux theory to identify key connections generating pathological activity, providing insights into disease progression and potential intervention points. The intricate STN‐GPe interplay is highlighted, shedding light on compensatory mechanisms within this circuitry may initially counteract changes but later contribute to pathological alterations as disease progresses. The framework addresses the need for comprehensive evaluation methods to assess intervention outcomes. Cross‐correlations between state variables provide superior early warning signals compared to traditional indicators relying on critical slowing down. By elucidating compensatory mechanisms and circuit dynamics, the framework contributes to improved management, early detection, risk assessment, and potential prevention/delay of PD development. This pioneering research paves the way for precision medicine in neurodegenerative disorders.

## Graphical abstract

This study presents a computational framework for understanding Parkinson's disease progression and potential interventions. The researchers developed a model of basal ganglia circuitry, revealing how compensatory mechanisms initially maintain normal function despite dopamine loss. They identified early warning signals for disease onset and evaluated intervention strategies. The approach offers insights into circuit dynamics, early detection, and targeted treatments for Parkinson's disease.

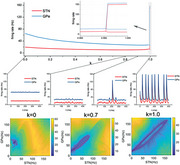

## 1 Introduction

PD represents a complex neurodegenerative disorder characterized by the progressive degeneration of dopaminergic neurons in the substantia nigra pars compacta, leading to dopamine depletion in the basal ganglia (BG).^[^ ^1^, ^2^, ^3^ ^]^ Normal brain function depends on the precise modulation of neuronal firing activity and spatiotemporal patterns, including oscillatory activity among neuronal networks.^[^ ^4^ ^]^ In PD, the progressive degeneration of dopaminergic neurons leads to intricate dysfunctions within the BG, resulting in persistent alterations in the firing rate and oscillatory synchronization among BG nuclei. which contribute to motor symptoms.^[^ ^1^, ^2^, ^3^, ^5^, ^6^, ^7^ ^]^ Motor symptoms such as bradykinesia, tremors, and rigidity are commonly used for diagnosis, but these signs become apparent only after a substantial loss of dopamine‐producing neurons has already occurred.^[^ ^3^, ^8^, ^9^ ^]^ This suggests the existence of a prolonged presymptomatic and premotor phase where compensatory mechanisms work to postpone the onset of disabling symptoms.^[^ ^2^, ^10^, ^11^ ^]^ As a result, the diagnosis of PD typically occurs after a considerable loss of dopaminergic neurons and the depletion of these compensatory mechanisms, complicating the effectiveness of interventions aimed at stopping or reversing the progression of the disease.

The potential to predict PD before the onset of motor symptoms could revolutionize disease management and pave the way for preventive interventions targeting the underlying circuit mechanisms. Identifying early warning signals and understanding the compensatory mechanisms within the BG circuitry are promising avenues for deciphering the pathophysiology of PD and discovering novel therapeutic targets. Advances in understanding complex systems and their critical transitions have illuminated the possibility of predicting disease onset and identifying early warning signals.^[^ ^12^, ^13^, ^14^, ^15^ ^]^ Critical transitions, characterized by abrupt shifts in the dynamics of complex systems, can occur as the system approaches a tipping point. Although traditionally detected through “critical slowing down,” where the system shows a reduced recovery rate following perturbations as it nears a bifurcation point, this may not be a universal indicator for all critical transitions, necessitating alternative approaches for early detection.

In this study, we propose a comprehensive theoretical framework utilizing the concept of non‐equilibrium dynamics in neural networks to identify early warning signals for critical transitions in PD. Our framework builds on the understanding that non‐equilibrium systems, such as neural networks, exhibit irreversible behaviors, characterized by the breaking of time‐reversal symmetry.^[^ ^16^, ^17^, ^18^, ^19^ ^]^ We hypothesize that specific behaviors in the cross‐correlations between the forward and backward directions of state variables within obtained time series can serve as reliable early warning signals for critical transitions in PD. Moreover, our framework addresses a critical gap in the field of neurodegenerative diseases: the lack of a comprehensive method to evaluate the effectiveness of interventions in the underlying disease process. Currently, assessing the impact of interventions on disease progression in PD is challenging due to the lack of reliable measures. By integrating the detection of early warning signals with the quantification of treatment outcomes, our framework aims to offer a holistic approach to precision medicine in PD.

The primary objectives of this study are threefold: first, to elucidate the circuit mechanisms, including compensatory mechanisms within the BG circuitry, to better understand the underlying pathophysiology of PD; second, to investigate the potential of our cross‐correlation approach in detecting early warning signals for critical transitions in PD; and third, to evaluate the effectiveness of interventions in restoring the system to its normal functional state at different stages of disease progression. To achieve these objectives, we constructed a circuit model and employed a simulated annealing optimization algorithm to identify the relevant parameters capable of replicating BG activity under both normal and Parkinsonian conditions. By introducing the non‐equilibrium landscape and flux framework, we identified key connections responsible for generating pathological activities. Our findings suggest that the cross‐correlation indicators can be detected earlier than traditional indicators based on critical slowing down. Our approach not only provides a practical tool for early detection but also facilitates the identification of at‐risk individuals and enables the evaluation of treatment effectiveness in the underlying disease process. Ultimately, this research could contribute to improved management strategies for PD, with the potential to prevent or delay the development of the disease.

## 2 Results

### 2.1 Neural Circuit Mechanism in Parkinson's Disease

**Figure 1:**
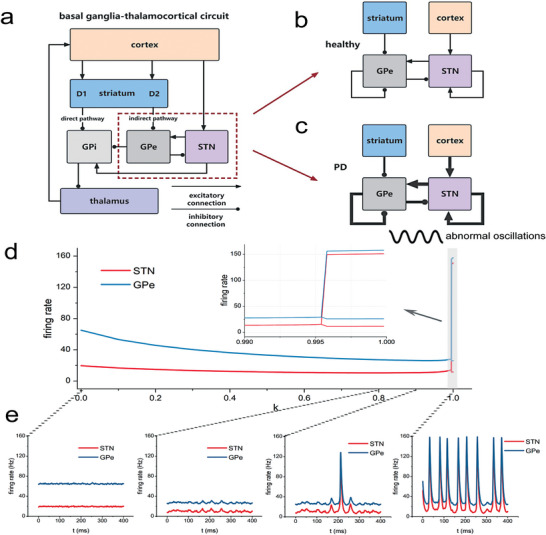
The STN‐GPe circuit diagram and the model results from the healthy to the Parkinsonian state. a–c) Major connections of the circuit model. Arrows denote excitatory connections and lines ending with circles denote inhibitory connections. The STN neurons serve as a significant origin of excitatory inputs to the globus pallidus externa (GPe), while the GPe neurons serve as a significant source of inhibitory inputs to the STN. Furthermore, our models incorporate a cortical circuit, where excitatory cortical neurons project to the STN. Using a simulated annealing optimization algorithm to search for the parameters that can reproduce the alterations in average firing rates and firing patterns observed in experimental settings between the healthy and pathological conditions. Our result suggest the emergence of beta‐band oscillations can be attributed to enhanced connection strengths in the STN‐GPe network (indicated by the thickened lines in (c)). d, e) illustrate that the model can accurately replicate the firing patterns and rates of healthy and pathological conditions. The healthy parameter set lacks oscillations, while the Parkinson's parameter set induces oscillations at around 13*Hz*. With the assumption of linearity in parameter changes, the firing rates of STN and GPe remain relatively stable until they reach a bifurcation point.

The basal ganglia comprise interconnected circuits essential for motor control, including the striatum, the globus pallidus (GP), the subthalamic nucleus (STN) and the substantia nigra (**Figure** **1**a). In a healthy brain, the direct pathway of the BG facilitates movement by inhibiting the output nuclei GPi (the internal segment of the globus pallidus), while the indirect pathway impedes movement through a sequence of inhibitory and excitatory projections that ultimately increase GPi output. PD disrupts this delicate balance, reducing the activity of the direct pathway and enhancing that of the indirect pathway. This imbalance promotes excessive inhibition of thalamic and cortical activity by GPi, resulting in the hallmark motor symptoms of PD.^[^ ^20^, ^21^, ^22^ ^]^

While changes in spike rates have traditionally been emphasized in models of BG dysfunction, recent studies have highlighted the importance of alterations in firing patterns, for instance, the excessive abnormal synchronized oscillatory activity within the beta frequency range (12‐30 Hz) across BG nuclei is strongly implicated in the motor symptoms of PD.^[^ ^22^ ^]^ Recent researches have elucidated multiple neural loops within the BG network capable of generating PD‐associated beta synchronization.^[^ ^23^, ^24^, ^25^, ^26^, ^27^ ^]^ The STN‐GPe network, in particular, has been identified as crucial in the generation of pathological rhythmic network activity. The activity pattern of this network is strongly correlated with motor function, and interventions targeting these abnormal activities have shown promise in alleviating motor dysfunction.

Building on this foundational knowledge, our study introduces a computational model of the STN‐GPe network, informed by experimental data from nonhuman primates and human PD patients.^[^ ^24^, ^25^, ^26^ ^]^ This model departs from previous efforts that focused on simulating individual neurons within specific architectures, which, while offering valuable clinical insights, lacked a holistic view of the system's dynamics. Our approach adopts a network perspective, utilizing the mean‐field methodology to provide a more comprehensive understanding of the underlying circuit mechanisms involved in PD. As illustrated in Figure 1b,c, our model identifies the STN neurons as a critical source of excitatory input to the GPe, with GPe neurons reciprocally providing inhibitory feedback to the STN. The model further incorporates a cortical circuit, with excitatory cortical neurons projecting to the STN, thereby influencing the systems dynamics. These dynamics are characterized by the firing rates of the STN and GPe nuclei, which are determined by both the electrophysiological properties (activation function) of each nucleus and the effective connection strengths between them.

#### 2.1.1 Parkinson's Disease Development Under the Influence of Compensatory Mechanisms

Identifying changes in network properties, specifically connection weights within the STN‐GPe network, is crucial for understanding how dopamine loss triggers the transition from normal to pathological activity. We constructed a cost function that quantifies the discrepancies between the simulation results of our model and the experimental data. To optimize these parameters minimizing this cost function, we employed a simulated annealing optimization algorithm, detailed in the Experimental Section.

The optimization results are presented in **Table** **1**, which lists two parameter sets, **w_h_** and **w_PD_**, corresponding to the healthy and Parkinsonian states, respectively. The simulations, depicted in Figure 1d, e, demonstrate the model's fidelity in reproducing the observed changes in average firing rates and firing patterns between healthy and pathological conditions. Under the healthy parameter set **w_h_**, the system achieves a stable state without oscillations at any specific frequency. Conversely, the Parkinsonian parameter set **w_PD_** induces oscillatory activity at approximately 13 Hz, a finding consistent with previous experimental and theoretical studies.^[^ ^24^, ^25^, ^26^, ^27^ ^]^ This emergence of beta‐band oscillations appears to result from enhanced connectivity within the STN‐GPe network and increased cortical excitability to the STN, potentially driven by dopamine loss, as both STN and GPe express D2 receptors that are modulated by dopamine.^[^ ^11^, ^28^, ^29^ ^]^

**Table 1:** Fitted model parameters.

Parameters	*w*_*SG*_	*w*_*SC*_	*w*_*GS*_	*w*_*GG*_	*Input*_*Str*_	*w*_*SS*_
w_h_	5.55	63.15	6.37	1.92	0.15	1.97
w_PD_	28.37	73.40	9.68	8.27	0.2	28.04

Understanding the progression of Parkinson's disease is essential for elucidating its pathogenesis. However, capturing the intermediate state between normal and pathological conditions remains challenging in experimental settings. To bridge this gap, we assumed a linear progression in parameter changes, represented as **w** = **w_h_** + *k*(**w_PD_** − **w_h_**), *k* ∈ [0, 1] is the progression value. This model allowed us to quantify alterations in firing activity within the circuit as the disease evolves. The phase diagram (Figure 1d) illustrates that the firing rates of STN and GPe remain relatively stable until reaching a bifurcation point (*k* = 0.995), which closely aligns with the fully established pathological state. This computational finding provides mechanistic insight into the observed clinical phenomenon of delayed symptom onset in PD, suggesting that compensatory mechanisms within the basal ganglia circuitry effectively maintain near‐normal firing patterns even as underlying neurodegeneration progresses significantly.^[^ ^2^, ^11^ ^]^

The increased STN‐GPe connectivity can be viewed as a consequence of dopamine loss. Alternatively, changes in the STN‐GPe circuit may serve as compensatory mechanisms. Studies have shown reduced autonomous activity in GPe following dopamine depletion.^[^ ^30^ ^]^ The enhanced excitatory input from the cortex to the STN and from the STN to the GPe may compensate for the reduced GPe activity. While these compensatory mechanisms aim to maintain balance, the suppressed direct pathway in the BG (striatum to GPi) under PD conditions challenges their efficacy. The excessive STN activity may trigger increased firing in the GPi through its direct excitatory projections, exacerbating the motor symptoms of PD. In response, the heightened inhibition of the GPe toward the STN attempts to modulate its activity, striving to maintain it within a proper range. This delicate balance reflects an ongoing compensatory effort within the basal ganglia circuitry to preserve normal output levels and potentially slow the onset of motor symptoms. However, as PD progresses, the capacity for compensation may be exceeded, leading to an intensification of STN‐GPe circuit interactions, which could inadvertently foster the development of pathological activities, such as beta‐band oscillations. Subsequent sections will proceed to explore in depth the extensive discussions regarding these topics.

### 2.2 New Therapeutic Insights from Neural Circuit Mechanism

The presence of compensatory mechanisms within neural circuitry allows for a significant degree of dopamine depletion in the initial stages of PD without the emergence of motor symptoms. This poses intriguing questions: Can early warning signals be detected during the presymptomatic phase of the disease, and can effective treatments be administered post or even pre‐clinical onset of PD motor symptoms?

The established existence of abnormal beta oscillations in PD^[^ ^23^, ^31^, ^32^, ^33^, ^34^, ^35^ ^]^ underscores the importance of understanding changes in connection weights within the circuit during the transition from a normal state to a PD state. This understanding is vital for elucidating the underlying pathogenic mechanism and identifying potential treatments. The power of these oscillations correlates closely with symptom severity, as demonstrated by the efficacy of treatments such as dopaminergic medications and deep brain stimulation (DBS) in reducing beta oscillation power and alleviating symptoms. To effectively mitigate abnormal oscillatory activity, interventions must be identified that can achieve the desired outcome.

While bifurcation analysis has been a common tool in previous studies to explore the effects of weight changes on oscillatory behavior within the basal ganglia,^[^ ^26^ ^]^ it is essential to consider the presence of noise in real‐world systems. Oscillations can manifest even within the monostable phase, and perturbations can induce transitions before reaching a bifurcation point. Stochastic shocks play a crucial role in triggering such transitions. Therefore, relying solely on bifurcation analysis may not fully capture the differences in stability among various intervention methods and quantitatively assess treatment efficacy for pathological oscillatory activity. Developing more specific and quantitative approaches is crucial to evaluate stability and intervention effectiveness accurately.

**Figure 2:**
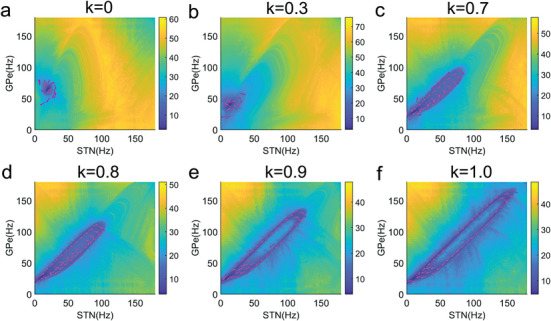
The underlying potential landscapes and fluxes for varying progression values. As the progression value *k* increases (indicating disease progression), this point attractor gradually becomes unstable and a stable limit cycle oscillatory attractor emerges. The magenta arrows indicate the non‐equilibrium flux part of the drive forces.

The concept of the attractor landscape has traditionally served as a visual representation to describe the functioning of complex systems such as neural circuits.^[^ ^36^ ^]^ However, these landscapes have often been depicted in illustrative forms.^[^ ^37^, ^38^, ^39^ ^]^ To address this limitation, we have developed a non‐equilibrium potential landscape and flux framework for general neural circuits, drawing inspiration from thermodynamics and statistical mechanics. This framework enables a quantitative assessment of the global stability of the system, including the stable state observed in healthy conditions and the emergence of oscillatory activities in pathological conditions. **Figure** **2** illustrates the underlying potential landscapes and fluxes for varying progression values *k*. At *k* = 0, a single‐point attractor representing the healthy state of the system is observed. As *k* increases, indicating disease progression, this point attractor gradually becomes unstable. When the progression value *k* approaches 1, a stable limit cycle oscillatory attractor emerges, marking a significant shift in the systems dynamics.

The dynamics of a non‐equilibrium system are governed by both the gradient of the potential landscape and the steady‐state probability flux.^[^ ^16^, ^19^, ^40^ ^]^ The gradient force acts to attract and stabilize the system toward the point attractor, while the rotational flux force tends to destabilize the point attractor, fostering the establishment of a stable limit cycle oscillatory attractor. Once the system is drawn into the closed oscillation ring, the flux becomes the dominant driving force for the non‐equilibrium dynamics. Consequently, the flux serves as a crucial measure for quantifying the stability of oscillations, with a larger flux indicating that coherent oscillations are less susceptible to external fluctuations.

To quantitatively evaluate the effectiveness of various intervention methods for mitigating pathological oscillatory activity, we have developed an error function. This function includes a term representing the magnitude of flux and another term measuring the discrepancy in firing rates observed in simulations relative to the normal state documented in experimental settings. A lower value of the error function signifies a closer approximation to the healthy state.

**Figure 3:**
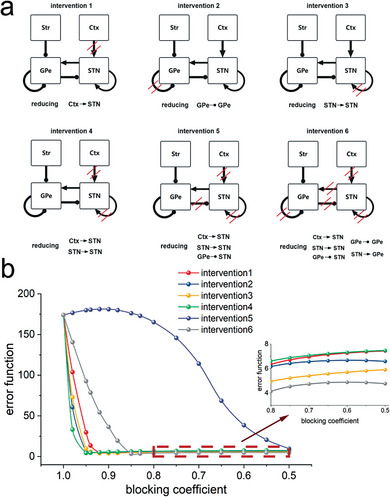
Evaluating the effectiveness of different intervention methods for addressing pathological oscillatory activity. a) The red slashes in each subfigure indicate the different modified connections within the STN‐GPe circuit for different intervention methods, respectively. b) Evaluating the treatment effectiveness of each intervention method through an error function with varied blocking coefficient on the corresponding targeted connections.

Our findings suggest that targeting the strengthened connections within the STN‐GPe circuit and the increased excitatory input from the cortex to the STN may be an effective strategy for restoring normal function in PD. To investigate potential intervention methods, we introduced a blocking coefficient γ ∈ [0, 1] to modulate specific connection weights by γ*w. **Figure** **3** presents the outcomes of different interventions, such as reducing the excitation from the cortex to the STN (*intervention*1) or reducing all primary connections in the STN‐GPe circuit that were strengthened due to dopamine depletion (*intervention*6).

Our study did not prioritize enhancing the inhibitory effect of GPe neurons as a primary factor in BG oscillations, as blocking ionotropic GABAergic transmission had minimal impact on GPe oscillations within the 8–15 Hz range, and in some cases even intensified them.^[^ ^24^ ^]^ Figure 3b displays the error function values following adjustments to various connections within the STN‐GPe circuit, as well as the input from the cortex. Our findings highlight the diverse effects of different manipulations in mitigating abnormal activities identified in the PD system. Certain interventions, such as interventions 1, 2, 3, and 4, which involve the excitation of STN, demonstrate immediate effects with relatively modest alterations observed in the respective connections. However, to achieve advanced treatment effectiveness, additional manipulations targeting specific sets of connections (e.g., intervention six, indicated by the gray line in the figure) may be essential.

#### 2.2.1 Early Warning Signal for the Onset of Pathological Oscillatory Activity in Parkinson's Disease

The limited effectiveness of current symptomatic treatments for PD can be attributed, at least in part, to the delayed diagnosis and initiation of therapy. Despite considerable efforts in the fields of neurology and psychiatry, there is currently no recommended diagnostic technology available for clinical use in detecting the early stages of neurodegenerative diseases. In complex systems characterized by numerous interacting components, abrupt state transitions, known as “critical transitions” are a common phenomenon.^[^ ^12^, ^13^ ^]^ The transition of the basal ganglia neural circuit from a normal to a pathological state, triggered by dopamine depletion, exemplifies such a critical transition. Previous research has often employed nonlinear dynamics and bifurcation theory to describe and predict these critical transitions. Critical slowing down a phenomenon generally observed near various types of tipping points has traditionally been regarded as an indicator of such transitions.^[^ ^12^, ^13^ ^]^ However, critical slowing down is not universally applicable as a warning signal for abrupt shifts, as signals based on this phenomenon can only be detected when the system is in close proximity to an approaching transition or bifurcation point.

**Figure 4:**
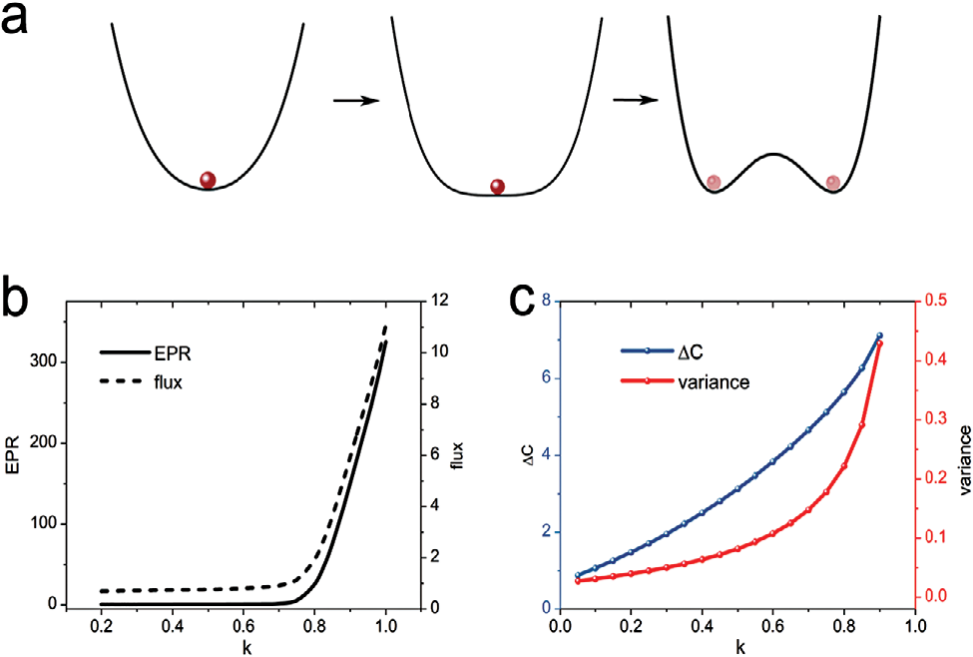
Early warning signals for the transition from the healthy state to the pathological state. a) The schematic of the attractor landscape of the system transitioning from a monostable to a limit‐cycle phase. b,c) Such transitions across a Hopf bifurcation are preceded by an increase in the entropy production rate, average flux, variance, and the differences in the cross‐correlations Δ*C*. c) shows that the increase in Δ*C* can be detected earlier than the one in the variance.

Our findings indicate the presence of a Hopf bifurcation point between the normal and pathological states as the disease progresses. As a potential consequence of critical slowing down, an increase in variance typically arises due to the shrinking basin of attraction and may be detected well in advance of a critical transition, as illustrated in **Figure** **4**a. Notably, the presence of noise can trigger oscillations, raising an intriguing question about the possibility of detecting crucial signals earlier than those suggested by previous studies based on critical slowing down.

As discussed earlier, an increase in the flux force can destabilize a stable state, providing a dynamical foundation for bifurcations or non‐equilibrium dynamical phase transitions. Figure 4b illustrates the increase in non‐equilibrium flux and its associated entropy production rate as the system progressively approaches the bifurcation point with an increasing progression value *k*. Notably, the quantification of non‐equilibrium flux and entropy production rate serves as measures of the degree of time‐reversal symmetry breaking in a non‐equilibrium system. Driven by these findings, we have identified distinctive patterns in the difference of cross‐correlations between the forward and backward temporal directions of the two state variables, denoted by Δ*C* in this study (details provided in the Experimental Section). These observed patterns serve as reliable indicators of the degree of time‐reversal symmetry breaking and offer early warning signals for predicting critical transitions.

Figure 4c further illustrates the upward trajectory of both the variance and Δ*C* as the system approaches the bifurcation point. Notably, the increase in variance becomes explicitly observable only after the progression value *k* > 0.5. In contrast, the evident increasing slope of Δ*C* can be detected earlier. Our research suggests that the early warning indicators derived from the cross‐correlation approach can be detected prior to those based on critical slowing down. In the broader context of understanding complex systems, unraveling the intricate mechanisms governing their behavior remains a significant challenge. Consequently, the cross‐correlation approach emerges as a more practical and accessible methodology for predicting critical transitions, relying solely on the time series data of the state variables. This approach enhances our ability to predict and potentially mitigate the impacts of critical transitions in complex systems.

#### 2.2.2 Early Intervention and Preventive Treatment for Parkinson's Disease

The potential for predicting PD before the onset of motor symptoms offers a promising avenue for developing preventive treatments and early interventions. By targeting the underlying circuit mechanisms in the earliest stages of the disease, it may be possible to slow or even halt disease progression. This approach represents a paradigm shift from current symptomatic treatments, which are often initiated after significant neurodegeneration has occurred. Our framework provides tools to evaluate morbidity risk, offer early warnings, and quantify treatment effectiveness.

**Figure 5:**
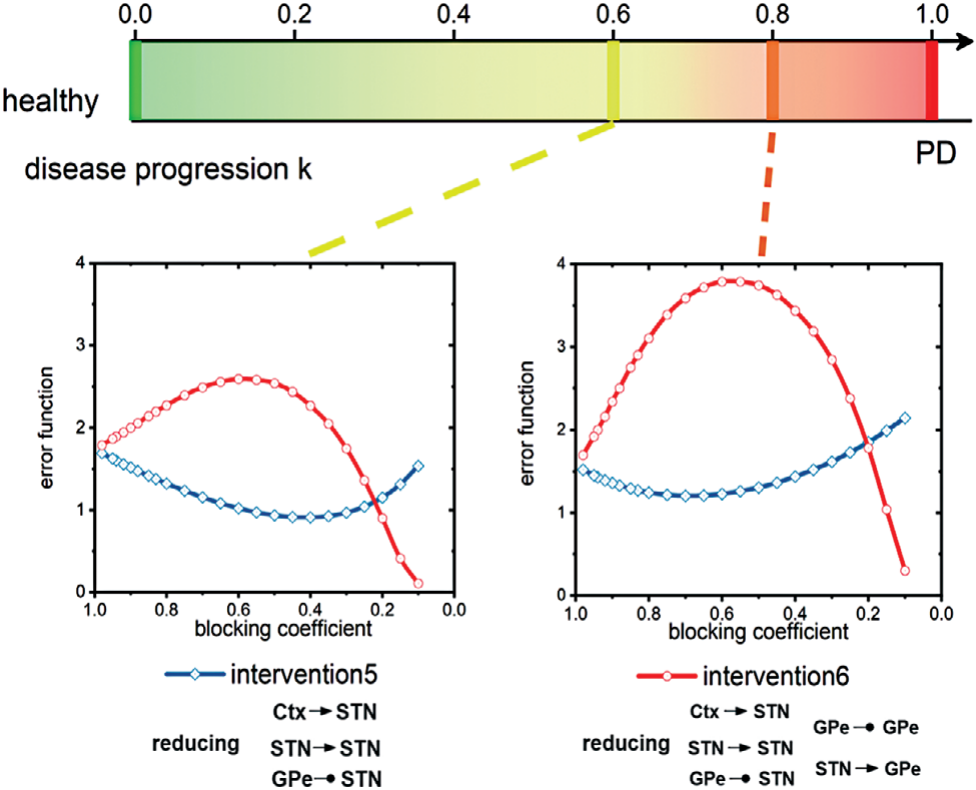
Evaluating the effectiveness of different intervention methods at the early stage of the disease progression. The intervention strategy aiming at reducing all augmented connections within the STN‐GPe circuit caused by dopamine depletion can successfully restored the system to its normal functional state, as indicated by the red curves.The timing of the intervention is crucial, with earlier interventions producing more favorable treatment outcomes.

Diverging from the treatment approach employed after the onset of pathological oscillatory neural activity, as depicted in Figure 3, we evaluated the therapeutic potential of interventions during the initial phases of disease progression. Here, we investigated the effects of interventions administered at two distinct time points (*k* = 0.6, 0.8), focusing on the efficacy of these treatments in the initial phases of disease progression. The results, depicted in **Figure** **5**, demonstrate that the intervention strategy labeled “Intervention 6”, aimed at reducing all enhanced connections within the STN‐GPe circuit due to dopamine depletion, was markedly effective in returning the system to its normal functional state, as indicated by the red curves. Importantly, our data reveal a positive correlation between the timing of intervention and the outcomes, with earlier interventions leading to more beneficial results. This observation suggests that interventions aimed at modulating system dynamics could potentially be applied earlier, offering a promising avenue for preemptive treatments in Parkinson's disease.

In this discussion, we limit our focus to intervention strategies 5 and 6, as the other strategies did not yield satisfactory effects. Previous discussions highlighted a set of compensatory mechanisms within this system designed to delay the onset of disease symptoms. Alternative intervention strategies, such as the weakening of a single synaptic connection, might interfere with these compensatory mechanisms, causing a further deviation from the system's normal neural activity. This underscores the importance of selecting early intervention methods with caution and precision, to avoid disrupting the delicate balance of neural compensatory mechanisms that delay the onset of PD symptoms.

## 3 Discussion

PD pathogenesis involves complex alterations in BG circuitry, characterized by persistent changes in firing rates and excessive synchronized oscillatory activity.^[^ ^1^, ^5^, ^6^, ^7^ ^]^ These changes underlie the motor symptoms that typically manifest only after substantial dopaminergic neuron loss, indicating a prolonged presymptomatic phase with active compensatory mechanisms.^[^ ^2^, ^3^, ^10^, ^11^ ^]^ Our study focuses on elucidating these compensatory processes and their eventual breakdown, which is crucial for developing more effective treatments.

To investigate the mechanisms underlying abnormal BG activity in PD, we examined neuroplasticity‐driven changes in internuclear connections. Using a circuit model and simulated annealing optimization, we identified parameters that accurately replicate BG activity in both normal and Parkinsonian states. This approach provides insights into the adaptive changes occurring within the BG circuit during disease progression and offers a framework for exploring potential therapeutic targets.

Our findings reveal that beta‐band oscillations in PD stem from increased connectivity within the STN‐GPe network and enhanced cortical excitation to the STN, consistent with previous studies.^[^ ^27^, ^41^, ^42^ ^]^ These changes may represent both direct consequences of dopamine depletion and compensatory mechanisms. The loss of dopamine affects D2 receptors in both STN and GPe,^[^ ^11^, ^28^, ^29^ ^]^ while the amplification of cortical and STN excitation may initially compensate for reduced GPe autonomous activity.^[^ ^30^ ^]^ However, this compensation may ultimately contribute to pathological activity. Excessive STN activation can increase GPi firing, further suppressing thalamic activity and exacerbating motor symptoms. As the disease progresses, these compensatory mechanisms become overwhelmed, leading to strengthened STN‐GPe connectivity and pathological oscillations.

These insights into circuit mechanisms offer potential targets for PD treatment. The increased connections within the STN‐GPe circuit present promising intervention points. Evaluating such interventions requires careful consideration of two key factors: quantifying the deviation from the healthy state and assessing the risk of inducing abnormal oscillations. Our approach provides a framework for developing more targeted and effective therapies for PD.

Our non‐equilibrium framework provides valuable insights into the dynamics of PD.^[^ ^16^, ^19^, ^40^ ^]^ To quantify these dynamics and evaluate potential interventions, we constructed an error function incorporating both the flux magnitude and the deviation from healthy state firing rates. Our results align with previous research while offering new perspectives on intervention strategies.^[^ ^24^, ^25^ ^]^ We found that inhibiting connections between the GPe and the STN, as well as blocking cortical input, can effectively eliminate beta oscillations. Notably, selectively targeting excitatory connections proves more efficient in abolishing oscillations compared to blocking all synaptic connections to the STN. Furthermore, we observed that reducing all connections augmented by dopamine loss brings the system closer to a healthy state, albeit with slightly diminished efficacy compared to more targeted interventions. These findings provide a nuanced understanding of potential therapeutic approaches for PD, highlighting the importance of targeted interventions in restoring normal basal ganglia function. The framework we've developed offers a quantitative method for evaluating the efficacy of various intervention strategies, potentially guiding the development of more effective treatments for PD.

Our theoretical interventions, while based on computational models, have significant potential for translation into real‐world applications. One prominent real‐world intervention is DBS,, which targets nuclei within the basal ganglia, such as the STN. However, the underlying mechanism of DBS remains unclear, whether it results from an increase or decrease of STN activity. The cell‐specific effects of both the pallidosubthalamic and reciprocal pathways are still not fully understood and require further investigation. Given these uncertainties, our model's insights may not directly optimize DBS parameters. Nevertheless, our work has implications for emerging technologies such as optogenetics and opto‐DBS, which allow for cell type‐ and projection‐specific modulation.^[^ ^43^, ^44^, ^45^ ^]^ These techniques, while currently limited to animal models, provide a platform for validating our theoretical predictions and potentially inspiring more precise therapeutic strategies. Additionally, pharmacological interventions can benefit from our findings. While current treatments like levodopa and dopamine agonists target the dopaminergic system, our model suggests the potential benefit of combination therapies that modulate multiple neurotransmitter systems. This could lead to more comprehensive management of PD symptoms.

Our study introduces a novel approach to early detection and intervention in PD, addressing the limitations of current symptomatic treatments. We propose a cross‐correlation method that exploits the inherent irreversibility of non‐equilibrium neural networks to identify early warning signals preceding significant dopaminergic neuron loss. This method analyzes variations in cross‐correlations between forward and backward temporal directions of state variables, revealing distinctive patterns associated with time‐reversal symmetry breaking in non‐equilibrium systems. These patterns serve as robust early indicators for predicting critical transitions, surpassing the predictive capabilities of methods based on “critical slowing down”.^[^ ^12^, ^13^ ^]^ Our approach offers a practical means of predicting critical transitions using only time series data of state variables, circumventing the need for detailed mechanistic understanding of complex systems. Furthermore, we explored the therapeutic implications of our circuit‐based framework, assessing the impact of interventions at various stages of disease progression. Our findings indicate that earlier interventions can more effectively restore normal functional states, aligning with experimental evidence showing reduced parkinsonian symptoms with early levodopa administration.^[^ ^3^, ^11^, ^46^ ^]^

The practical implementation of our early intervention detection system for PD presents both challenges and opportunities. Our approach, which relies on analyzing non‐equilibrium dynamics in neural networks, requires high‐precision temporal data of neural activity that is currently challenging to obtain non‐invasively. To address this limitation, we propose a two‐step process. First, we utilize existing non‐invasive methods such as neuroimaging (MRI, PET scans) and cerebrospinal fluid (CSF) biomarkers to identify individuals at high risk of developing PD.^[^ ^47^, ^48^, ^49^ ^]^ These techniques have demonstrated efficacy in predicting motor progression and detecting early structural and functional brain changes. For high‐risk individuals, we then propose the use of implantable microelectrode arrays (MEAs) to collect time‐accurate neural activity data from critical brain regions like the GPe and STN. Recent advancements in MEA technology, including improved biocompatibility and durability, allow for high‐density, long‐term recording of neural activity with minimal tissue damage.^[^ ^50^, ^51^, ^52^ ^]^ These arrays can be specifically targeted to basal ganglia regions, providing detailed insights into neural oscillatory patterns. Additionally, local field potential recordings from implantable devices offer a broader view of neural dynamics. While this approach involves invasive procedures, it provides the necessary temporal precision for our early warning detection system, potentially enabling intervention before the onset of motor symptoms. This strategy balances the need for accurate data with patient safety and comfort, representing a promising path forward in early PD detection and intervention.

In conclusion, our framework addresses critical gaps in PD research by offering the ability to evaluate morbidity risk, provide early warning signals, and quantify treatment effectiveness. This comprehensive approach could significantly improve PD management strategies, potentially leading to preventive interventions and more effective treatments at various stages of disease progression.

## 4 Experimental Section

### Computational Model

Historically, PD pathophysiology studies focused on dopamine loss and its effects on basal ganglia / thalamocortical circuit function, with changes in neuronal firing rates considered central to parkinsonism.^[^ ^20^, ^21^ ^]^ However, the expected rate changes along direct and indirect pathways were not consistently observed, suggesting a more complex pathology.^[^ ^5^, ^22^, ^53^ ^]^ This inconsistency had led to a shift in focus toward changes in firing patterns, particularly abnormal oscillatory activities within the basal ganglia and related nuclei. Beta‐band oscillatory activity within the basal ganglia had emerged as a significant biomarker for PD symptoms.^[^ ^54^, ^55^, ^56^ ^]^ Numerous studies had demonstrated a positive correlation between the amplitude of these oscillations and symptom severity, with evidence suggesting that modulating beta oscillations could alleviate motor symptoms in PD patients.^[^ ^57^, ^58^, ^59^, ^60^ ^]^ While not universally present in all PD cases, reflecting the disease's heterogeneity, beta oscillations remain a valuable subject for investigation, particularly in modeling neural network dynamics. The relationship between these oscillations and the chronological onset of motor symptoms, as well as their connection to non‐motor symptoms, remains an active area of research. However, studies suggest a possible link between beta‐band oscillatory activity and non‐motor aspects of PD.^[^ ^56^, ^61^ ^]^

In the computational modeling of biological systems, the necessity of simplification was acknowledged to render complex problems tractable while retaining the essential system features. The model was predicated on the hypothesis that beta band oscillatory activity could serve as a dependable biomarker for PD. The model's focus on the STN loop stems from the theory that beta oscillations originate in this loop due to its “pacemaker‐like” properties, supported by experimental evidence demonstrating oscillations in vitro within the GPe and STN in isolation.^[^ ^62^ ^]^ Blocking STN‐GPe connections eliminated excessive beta oscillations, and glutamatergic inputs from the motor cortex to the STN during dopamine depletion may contribute to pathological oscillations.^[^ ^24^ ^]^ To simplify the cortico‐basal ganglia‐thalamo‐cortical loop, the net effects of removed nuclei was integrated, such as the motor cortex, striatum, GPi, and thalamus, by introducing effective inputs to the STN‐GPe circuit. This approach was underpinned by findings indicating that beta oscillations persist in the GPe‐STN loop even without striatum‐to‐GPe input, and the average neural firing activity of the motor cortex remained essentially constant.^[^ ^24^, ^63^ ^]^ Figure 1 illustrates the configuration of the models.

In the field of Parkinson's disease research, previous models had primarily focused on individual neurons and their specific connection architectures, resulting in limited understanding of the system as a whole. This approach to modeling the BG network was in line with larger‐scale approaches such as the Wilson and Cowan/Dayan and Abbot firing rate method or the mean‐field model. This approach, akin to the mean‐field approximation in statistical physics, allowed to study the macroscopic phenomena of the system without needing to know the detailed state of each microscopic component.^[^ ^64^, ^65^ ^]^

This model describes the dynamics of the circuit with the following firing rate equations:

(1) $$\tau_{S}\frac{dx_{S}}{dt}=-x_{S}+f_{S}\left(Input_{S}\right)$$

(2) $$\tau_{G}\frac{dx_{G}}{dt}=-x_{G}+f_{G}\left(Input_{G}\right)$$

where *x*_*S*_, *x*_*G*_ are the average firing rates of the STN and GPe nuclei, respectively. The activation functions, *f*_*S*_ and *f*_*G*_, are determined by the input to each population, represented by

(3) $$Input_{S}=w_{SS}x_{S}-w_{SG}x_{G}+w_{SC}Crx$$

(4) $$Input_{G}=w_{GS}x_{S}-w_{GG}x_{G}+Input_{Str}$$

(5) $$f_{i}\left(input_{i}\right)=\frac{M_{i}B_{i}}{\left(M_{i}-B_{i}\right)e^{\frac{-4input_{i}}{M_{i}}}+B_{i}},\;i=STN\;\mathrm{and}\;GPe$$

where *M*_*i*_ represents the maximum firing rate of each population and *B*_*i*_ represents the population firing rate in the absence of input. The synaptic weights, denoted as *w*_*ij*_, represent the synaptic weights of the connections from population *j* to population *i*. The motor cortex firing rate was set to a constant value, *Crx*, and inhibitory input from the striatum was represented by *Input*_*Str*_. All parameters, except for connection weights, were estimated directly from experimental data^[^ ^24^, ^25^, ^26^, ^63^ ^]^ (**Table** **2**). By adopting a network perspective and utilizing a mean‐field approach, this model provides a more comprehensive understanding of the circuit mechanism responsible for PD. This approach may had important implications for the development of novel therapeutic interventions for this debilitating disease.

**Table 2:** Parameter values derived from experimental data.

*M*_*S*_	*M*_*G*_	*b*_*S*_	*b*_*G*_	τ_*S*_	τ_*G*_	*Crx*	*PDFre* ^*e*^
300/*s*	400/*s*	25/*s*	65/*s*	6*ms*	4*ms*	4.8/*s*	13.7*Hz*
$H_{STN}^{e}$	$H_{GPe}^{e}$	$PD_{STN}^{e}$	$PD_{GPe}^{e}$	$H_{STN}^{\sigma }$	$H_{GPe}^{\sigma }$	$PD_{STN}^{\sigma }$	$PD_{GPe}^{\sigma }$
27.3/*s*	41.2/*s*	19.7/*s*	65.2/*s*	9.5/*s*	25.9/*s*	10.5/*s*	22.4/*s*

The parameters in Table 1 were obtained using a simulated annealing optimization algorithm to minimize the discrepancy between the model output and the experimental data. There were six parameters were optimized: *w*_*SG*_, *w*_*GS*_, *w*_*SC*_, *w*_*SS*_, *w*_*GG*_, and *input*_*Str*_. For each set of parameters, the optimization algorithm solved the firing rate equations numerically, using the ode23tb function in MATLAB, and the average firing rates and oscillation frequency were calculated. These simulated data are compared with experimental data using a cost function, given by

(6) $$\begin{array}{cc} cost\;function=\; & \sum_{i=S,G}{\left(\frac{H_{i}^{e}-H_{i}^{s}}{H_{i}^{\sigma }}\right)}^{2}+\sum_{i=S,G}{\left(\frac{PD_{i}^{e}-PD_{i}^{s}}{PD_{i}^{\sigma }}\right)}^{2}\\ & +\left({\frac{PDFre^{e}-PDFre^{s}}{8}}^{2}\right) \end{array}$$

$H_{i}^{e}$ and $H_{i}^{s}$ are the healthy average firing rates derived from the experimental data and our simulated results, respectively. Similarly, $PD_{i}^{e}$ and $PD_{i}^{s}$ correspond to the parkinsonian condition. $H_{i}^{\sigma }$ and $PD_{i}^{\sigma }$ are the associated standard deviations that can also be taken from experimental data. *PDFre* represents the oscillatory frequency in the parkinsonian condition. Notably, previous experimental evidences showed that a lesion of striatal input elevated the mean firing rate of GPe populations while beta oscillation power remained constant.^[^ ^24^ ^]^ Although this effect was not directly included in the cost function's constraints, a constraint was imposed that the model's oscillation amplitude must not decrease after the blockade of striatal input to GPe neurons and ensured that the amplitudes of oscillations in both STN and GPe remain consistent. Moreover, the cost function also incorporated a rigorous penalty for parameters that could still generate oscillations after the connection between STN and GPe was severed, serving as a constraint for the optimization process. All the weights were allowed to vary in a large range: [10^−2^, 100]. MATLABs sa.m program was used from the optimization toolbox. The algorithm ends only when the error function was below 1.

An error function had been created to assess the efficacy of various intervention techniques in managing pathological oscillations:

(7) $$error\;function=J^{2}+\sum_{i=S,G}{\left(\frac{H_{i}^{e}-x_{i}^{s}}{H_{i}^{\sigma }}\right)}^{2}$$

This function included a flux magnitude term and a firing rate discrepancy term, which compare simulation results to the healthy state in experimental settings. **J** represents the average non‐equilibrium flux of the system. $x_{i}^{s}$ represents the simulated firing rate of STN or GPe. A lower error function value indicated a better approximation to the normal state.

### Non‐Equilibrium Landscape and Flux Framework for General Neural Circuits

The attractor landscape metaphor was widely used to describe cognitive functions such as associative memory retrieval, classification, et al.^[^ ^36^ ^]^ However, such attractor landscape could only be explicitly quantified in limited specific networks, e.g., the symmetrical neural circuit in the original Hopfield model. To address the issues of global stability, a non‐equilibrium potential landscape and flux theory was developed for the general neural networks.^[^ ^16^ ^]^ The focus was on the probabilistic evolution of the neural network rather than following the individual trajectories for characterizing the dynamics globally. Due to the stochastic nature of a realistic neural network, the stochastic dynamics of the neural network could be described by a set of Langevin equations: $\frac{dx}{dt}=F\left(x\right)+\zeta$. ζ represents the stochastic fluctuations, which were assumed to follow a Gaussian distribution with autocorrelations specified by $\left\langle \zeta \left(x,t\right)\zeta \left(x,t^{\prime }\right)\right\rangle =2D\left(x\right)\delta \left(t-t^{\prime }\right)$. Here, **D**(**x**) is the diffusion coefficient matrix giving the magnitude of the fluctuations. δ(*t*) is a delta function. These stochastic differential equations could be mapped onto an equivalent Fokker‐Planck equation for the probability density: ∂*P*(**x**, *t*)/∂*t* = −∇ · (**F**(**x**)*P(**x**, *t*)) + ∇ · (∇ · (**D** *P*(**x**, *t*))). Furthermore, the steady state probability distribution *P*_*ss*_ can be solved from ∇ · (**F**(**x**)*P_*ss*_(**x**)) − ∇ · (∇ · (**D** *P*_*ss*_(**x**))) = 0. And then the potential landscape could be quantified by the relationship *U* = −*lnP*_*ss*_(**x**) analogous to equilibrium statistical mechanics, where the potential landscape was related to the equilibrium distribution through the Boltzman law.

In contrast to equilibrium systems, where the dynamics were governed solely by the gradient of the underlying energy function resulting in a zero net flux, the dynamics of non‐equilibrium systems were influenced by both the potential gradient and the curl flux. The non‐equilibrium potential *U* serves as a useful tool for characterizing the global behavior of such systems.

In a steady state, non‐equilibrium thermodynamic systems were associated with continuous entropy production. While the entropy of a non‐equilibrium system in steady state remains constant over time, there was a flow of entropy to the surroundings equal to the entropy generated spontaneously within the system. To calculate the entropy production, the entropy associated with the time‐dependent probability distribution could be focused on. Using the Fokker‐Planck equation and the definition of probability flux **J**(**x**), the time derivative of the system entropy *dS*/*dt* can be expressed as a sum of two terms:

(8) $$\frac{d}{dt}S\left(t\right)=\int dx\left(J\cdot {\left(DD\right)}^{-1}\cdot J\right)/P-\int dx\left(J\cdot {\left(DD\right)}^{-1}\cdot \left(F-D\nabla D\right)\right)$$

Notice that the first term on the right hand side of the equation was always larger or equal to zero due to the positive definite diffusion matrix **D**. Therefore, it could be identified as the total entropy production rate, which was the global thermodynamic dissipation or cost directly linked to the non‐equilibrium flux from the dynamics:^[^ ^40^ ^]^

(9) $$EPR={\dot{S}}_{tot}=\int dx\left(J\cdot {\left(DD\right)}^{-1}\cdot J\right)/P$$

The second term on the right hand side of the equation could then be regarded as the entropy flux from the system to the environment:

(10) $${\dot{S}}_{env}=\int dx\left(J\cdot {\left(DD\right)}^{-1}\cdot \left(F-D\nabla D\right)\right)$$

The total entropy change was equal to the sum of the entropy change of the system and that of the environment, leading to the emergence of the generalized non‐equilibrium first law of thermodynamics:

(11) $${\dot{S}}_{tot}=EPR=\dot{S}+{\dot{S}}_{env}$$

The entropy change of the non‐equilibrium system (${\dot{S}}_{sys}=\dot{S}$) could either be increased or decreased due to the entropy flow to the environments, while the total entropy change of the system and the environments, represented by the entropy production rate, was always non‐negative. This leads to the generalized non‐equilibrium thermodynamic second law:

(12) $${\dot{S}}_{tot}\geq 0$$

Many natural systems, including biological systems, were non‐equilibrium systems coupled to their environment. In these systems, the non‐equilibrium flux leads to time‐reversal symmetry breaking, as recent studies had shown. The degree of time‐irreversibility or detailed balance breaking could be measured by the cross correlation difference between the forward and backward directions in time. Unlike equilibrium systems, where both cross‐correlation functions were equal, non‐equilibrium systems show asymmetry in cross‐correlation. The driving force in non‐equilibrium systems tends to destabilize the current state toward a bifurcation point, which could be reflected in the changes of the non‐equilibrium flux and cross correlation between the forward and backward directions in time.

To calculate the cross correlation difference, the Langevin equations were first solved for two state variables of a non‐equilibrium system, such as the activities in the excitatory and inhibitory subpopulations in the Wilson‐Cowan model. The time series was obtained and the cross correlation difference, Δ*C*(τ) = 〈*x*_*E*_(0)*x*_*I*_(τ)〉 − 〈*x*_*I*_(0)*x*_*E*_(τ)〉 was calculated, for each time delay τ. The average Δ*C* was then calculated, which was no longer a function of time delay, by integrating over the length of the dynamical trajectory in time (*t*). This process was repeated for ten thousand times to obtain the final Δ*C* by averaging the results, given the stochastic nature of the system.

To test if changes in Δ*C* can be used as an early warning signal for critical transitions, time series data describing the destabilization of the current state approaching a bifurcation or transition point was required. For example, if the goal was to predict the transition from the “up” state to the “down” state in the Wilson‐Cowan model, time series data of the excitatory and inhibitory subpopulations need to be collected as the control parameter *I*_*E*_ is decreased. The corresponding Δ*C* can be calculated for different values of *I*_*E*_ and observe the trends of Δ*C* as the system approaches a transition point. The results suggest that cross correlation was closely related to non‐equilibrium flux and entropy production rate, and could serve as a practical early warning signal for predicting critical transitions, detecting them much earlier than the actual transition point.

## Conflict of Interest

The authors declare no conflict of interest.

## Data Availability

The data that support the findings of this study are available from the corresponding author upon reasonable request.
